# ROS-implicated apoptosis in *Candida albicans*: mechanistic insights into Aureobasidin A's antifungal activity

**DOI:** 10.3389/fmicb.2026.1725921

**Published:** 2026-02-06

**Authors:** Jiaxin Yi, Qinghua Zhang, Hao Zhou, Wei Fei, Juan Liao, Yi Huang, Jun Guo

**Affiliations:** 1 Department of Stomatology, University of Electronic Science and Technology of China, Chengdu, Sichuan, China; 2 Department of Stomatology, Sichuan Provincial Academy of Medical Sciences, Affiliated Hospital to School of Medicine, Sichuan Provincial People's Hospital, University of Electronic, Science and Technology of China (UESTC), Chengdu, Sichuan, China; 3 School of Clinical Medical, Chengdu Medical College, Chengdu, Sichuan, China; 4 Department of Stomatology, The First Affiliated Hospital of Chengdu Medical College, Chengdu, Sichuan, China; 5 Department of Stomatology, Sichuan Academy of Medical Sciences & Sichuan Provincial People's Hospital (Wenjiang Campus), Chengdu, Sichuan, China

**Keywords:** antifungal, apoptosis, Aureobasidin A, *C. albicans*, reactive oxygen species

## Abstract

**Objectives:**

Aureobasidin A (AbA) is a natural antifungal lipopeptide known to inhibit inositol phosphorylceramide (IPC) synthase. While its antifungal effect, mechanism via the inositol pathway involved in sphingolipid synthesis, and influence on ABC efflux pumps have been reported previously, its potential role in inducing programmed cell death and efficacy against oral candidiasis remain unexplored. This study aimed to elucidate a novel, complementary mechanism of AbA against *Candida albicans* (*C. albicans*), focusing on ROS-implicated apoptosis, and to evaluate its therapeutic potential for oral candidiasis.

**Methods:**

*In vitro* experiments were initially conducted to assess the inhibitory effects of AbA on the virulence factors of *C. albicans* and investigate its impact on intracellular reactive oxygen species (ROS) levels and mitochondrial function to infer its potential apoptotic pathways. Subsequent transcriptome sequencing (RNA-seq) was employed to explore apoptotic mechanisms, with key genes validated by qRT-PCR. Finally, a murine oral candidiasis model was established to evaluate its *in vivo* antimicrobial activity and explore its clinical translational potential.

**Results:**

AbA potently inhibited the growth and key virulence of *C. albicans*. Against strain SC5314, its minimum inhibitory concentration (MIC) was 0.0625 μg/mL, with 75% fewer colonies at 72 h. After 4 h treatment, intracellular reactive oxygen species (ROS) increased by 2.75-fold, and propidium iodide (PI) fluorescence confirmed apoptosis induction. RNA-sequencing (RNA-seq) showed activation of oxidative stress-related pathways, validated by qRT-PCR: oxidative stress genes (*TSA1, NADPH* oxidase*, MCA1, CAT1*) were significantly downregulated. These findings suggest that AbA induces apoptosis, a process critically mediated by the activation of the oxidative stress pathway. In murine models, 1-week topical AbA reduced tongue fungal burden by 80%, inflammatory cell infiltration area by 60%, and alleviated tongue pathological damage.

**Conclusion:**

Beyond its known effect on sphingolipid synthesis, AbA exerts potent antifungal effects, which our data suggest involve the induction of ROS accumulation and subsequent mitochondrial dysfunction, leading to apoptosis. This dual mechanism highlights its promise as a therapeutic candidate, especially against azole-resistant infections.

## 1 Introduction

Natural products are a rich source of antifungal agents due to their structural diversity and biological specificity (Chopra and Dhingra, 2021). Aureobasidin A (AbA), a cyclic lipopeptide antibiotic derived from *Aureobasidium pullulans*, has garnered interest for its potent activity and low toxicity (Zhen et al., 2022). Its established primary mechanism involves the specific inhibition of inositol phosphorylceramide (IPC) synthase, a key enzyme in sphingolipid biosynthesis, disrupting membrane integrity (Mota Fernandes and Del Poeta, 2020; Khalfe and Rosen, 2022). Notably, AbA-mediated IPC synthase inhibition can also interfere with the function of membrane-localized ABC efflux pumps (e.g., *CDR1, CDR2*), which are major mediators of azole resistance in *C. albicans*. However, the complete spectrum of AbA's antifungal actions, particularly its potential to induce programmed cell death (PCD) and its efficacy in treating oral candidiasis, remains inadequately explored.

Apoptosis, a form of PCD, is characterized by conserved morphological changes including cell shrinkage, chromatin condensation, and DNA fragmentation (Obeng, 2021). It can be initiated through intrinsic (mitochondrial) or extrinsic (death receptor) pathways (Bertheloot et al., 2021). The intrinsic pathway is often precipitated by cellular stress, leading to mitochondrial outer membrane permeabilization, release of cytochrome c (Cyt C), and activation of caspase proteases (Zhou et al., 2024). Notably, fungi, including *Candida albicans (C. albicans)*, possess a rudimentary apoptotic machinery that can be activated by external stimuli, presenting a novel target for antifungal development (Yuan and Ofengeim, 2024; Rodríguez-González and Gutiérrez-Kobeh, 2023).

Mitochondria are central regulators of this intrinsic apoptosis (Al Amir Dache and Thierry, 2023). Beyond their role in energy production, they are a primary source of reactive oxygen species (ROS; Palma et al., 2024). Excessive ROS accumulation causes oxidative damage, leading to mitochondrial dysfunction, loss of mitochondrial membrane potential (MMP), and the release of pro-apoptotic factors, thereby initiating a caspase-dependent apoptotic cascade (Chen et al., 2022). This ROS-mediated apoptosis is increasingly recognized as a critical mechanism of action for several antifungal agents (Vringer and Tait, 2023; Semighini et al., 2006).

Given that AbA's known action on membrane lipids could potentially generate secondary oxidative stress, we hypothesized that ROS-mediated mitochondrial apoptosis might be involved in its antifungal efficacy (Yaakoub et al., 2022; Atriwal et al., 2021). To date, AbA has not been approved for clinical use in the treatment of oral candidiasis, despite its potent *in vitro* antifungal activity. This study aims to systematically investigate this novel mechanism of AbA. By combining phenotypic assays, transcriptional profiling, and molecular analysis, we delineate how AbA induces ROS accumulation, mitochondrial damage, and subsequent apoptosis in *C. albicans*. Furthermore, we evaluate the *in vivo* therapeutic efficacy of AbA in a murine model of oral candidiasis, providing a comprehensive rationale for its development as a multi-mechanistic antifungal therapy for both oral and invasive infections.

## 2 Materials and methods

### 2.1 Strains, culture conditions, and reagents

The standard wild-type *C. albicans* strain SC5314 (Biobw, China) was used exclusively throughout this study. Strains were stored at −80 °C in 25% sterile glycerol. For routine cultivation, yeast extract-peptone-dextrose (YPD) medium (1% yeast extract, 2% peptone, 2% dextrose; Sigma, China) was used. For solid media, 2% agar was added. To activate the strain, frozen stocks were streaked onto YPD agar plates and incubated at 30 °C for 48 h. A single colony was then inoculated into YPD liquid medium and incubated overnight at 30 °C with constant shaking at 200 rpm. This overnight culture was diluted to an OD600 of 0.1 in fresh YPD medium and grown to the mid-logarithmic phase (OD600 ≈ 0.6–0.8) at 37 °C with shaking for all experiments, ensuring a predominantly yeast-form population for consistent experimental conditions.

### 2.2 Assessment of antifungal efficacy

#### 2.2.1 Determination of minimum inhibitory concentration (MIC)

The MIC of AbA against *C. albicans* SC5314 was determined in accordance with the Clinical and Laboratory Standards Institute (CLSI) M27-A3 guidelines for broth microdilution. Briefly, AbA was first dissolved in methanol and then serially diluted two-fold in RPMI-1640 medium (with L-glutamine, without bicarbonate, buffered to pH 7.0 with 0.165 M MOPS; Sigma, China) across a 96-well microtiter plate. The final concentrations ranged from 0.0625 to 2 μg/mL. Each well was inoculated with 100 μL of a *C. albicans* cell suspension adjusted to 1 × 103 CFU/mL. The plate was incubated at 37 °C for 48 h. The MIC was defined as the lowest concentration of AbA that resulted in complete visual inhibition of growth. The MIC value was determined to be 0.0625 μg/mL, which was used for all subsequent experiments.

#### 2.2.2 Time-kill kinetics assay

*C. albicans* cells in the logarithmic growth phase were treated with AbA at concentrations of 0.0625 (1 × MIC), 0.125 (2 × MIC), 0.25 (4 × MIC), and 0.5 (8 × MIC) μg/mL. Aliquots were withdrawn at 0, 2, 4, 8, and 12 h, serially diluted in sterile phosphate-buffered saline (PBS; Gibco, USA), and plated on YPD agar in triplicate. Colony-forming units (CFUs) were enumerated after 48 h of incubation at 37 °C. Time-kill curves were plotted as log10 CFU/mL vs. time.

#### 2.2.3 Crystal violet biofilm assay

The effect of AbA on early biofilm formation was assessed using a crystal violet staining method. *C. albicans* cell suspension (1 × 106 CFU/mL in RPMI-1640) was added to a 96-well plate (200 μL/well). AbA was added to reach final concentrations of 0.0625–0.5 μg/mL. The plate was incubated at 37 °C for 2 h. After incubation, non-adherent cells were removed by gently washing twice with PBS (Gibco, USA). The adherent cells (biofilms) were fixed with methanol for 15 min, air-dried at room temperature, and stained with 0.1% crystal violet for 15 min. Excess stain was removed by washing with deionized water. The bound dye was solubilized with 95% ethanol, and the optical density (OD) was measured at 570 nm using a microplate reader (Thermo Fisher Scientific, USA).

#### 2.2.4 Extracellular polymeric substance (EPS) assay

The phenol-sulfuric acid method was used to quantify extracellular polysaccharide content (Yue et al., 2022). *C. albicans* cell suspension (1 × 106 CFU/mL) was co-cultured with AbA (0.0625–0.5 μg/mL) in Sabouraud dextrose broth (Sigma, China) for 24 h at 37 °C. The culture supernatant was collected by centrifugation at 3,000 × g for 3 min at 4 °C. To 200 μL of supernatant, 200 μL of 5% (w/v) aqueous phenol and 2 mL of concentrated sulfuric acid were added. The mixture was gently vortexed immediately after addition and incubated at room temperature in the dark for 1 h, and the absorbance was measured at 490 nm. Glucose was used to generate a standard curve.

#### 2.2.5 Adhesion assay

Mouse fibroblast L929 cells were used as the adhesion surface (Ibrahim et al., 2021). Cells were seeded in a 24-well plate at a density of 1 × 105 cells/well and cultured for 24 h to form a confluent monolayer. The monolayer was infected with *C. albicans* cell suspension (1 × 106 CFU/mL) at a multiplicity of infection (MOI) of 10:1 (*C. albicans* cells to L929 cells) and incubated for 5 h. Subsequently, AbA (0.0625–0.5 μg/mL) was added and incubation continued for another 2 h. Non-adherent fungal cells were removed by gentle washing three times with PBS. The adherent fungi were imaged under a light microscope, and the area of adhesion was quantified using ImageJ software (NIH).

#### 2.2.6 Hyphal inhibition assay

The ability of AbA to inhibit the yeast-to-hypha transition was evaluated in both liquid and solid media. For liquid assays, *C. albicans* cell suspension (1 × 106 CFU/mL) was incubated in Spider medium (1% nutrient broth, 1% mannitol, 0.2% K_2_HPO4) with AbA (0.03125 (1/2 × MIC), 0.0625 (1 × MIC), and 0.125 (2 × MIC) μg/mL) at 37 °C for 24 h. Hyphal formation was observed under an inverted light microscope, and hyphal length was quantified by analyzing five random fields per sample using ImageJ software. For solid assays, 50 μL of *C. albicans* suspension (1 × 105 CFU/mL) was spread onto Spider agar plates (supplemented with an additional 2% agar compared to the liquid Spider medium) containing the same concentrations of AbA. Plates were incubated at 37 °C for 4 days, and colony morphology was photographed.

### 2.3 Investigation of antifungal mechanism

#### 2.3.1 Cell membrane integrity and permeability assay

The integrity of the cell wall and membrane was assessed using crystal violet uptake and propidium iodide (PI; Beyotime, China) staining (Venkataraman et al., 2023; Luo et al., 2022). For crystal violet uptake, log-phase *C. albicans* cells (1 × 107 CFU/mL) were treated with AbA (0.5 μg/mL) for 10 h, stained with 5% crystal violet for 20 min, fixed with 10% tannic acid (Thermo Fisher, USA), and observed under a microscope. The percentage of cells with compromised walls was calculated from counts of 100 random cells. For PI staining, washed twice with PBS, then incubated with 5 μg/mL PI solution (Beyotime, China) in the dark for 30 min. Cells were visualized using confocal microscopy, and fluorescence intensity was quantified with a microplate reader, with five replicate wells per group.

#### 2.3.2 Scanning electron microscopy (SEM)

*C. albicans* cells treated with and without AbA (0.5 μg/mL) were fixed overnight in 2.5% (v/v) sterile glutaraldehyde at 4 °C, then washed three times with PBS (pH 7.4) to remove excess fixative. The samples were then dehydrated through a graded ethanol series (70%, 80%, 90%, 100%; 5 min each), critical-point dried, and sputter-coated with gold. The morphological changes were observed at magnifications of 2,000 × , 2,500 × (for overall cellular morphology) and 20,000 × (for surface ultrastructure) using a scanning electron microscope (Thermo Fisher, USA).

#### 2.3.3 Intracellular ROS detection

Intracellular ROS levels were measured using the fluorescent probe 2′,7′-dichlorodihydrofluorescein diacetate (DCFH-DA; Beyotime, China; Benedetti et al., 2022). *C. albicans* cells (1 × 106 CFU/mL) treated with AbA (0.0625–0.5 μg/mL) for 4 h were collected by centrifugation at 5,000 × g for 5 min at 4 °C, washed with PBS, and incubated with 10 μM DCFH-DA at 37 °C in the dark with gentle shaking for 30 min. The cells were washed again to remove excess probe. Fluorescence images were captured using a confocal laser scanning microscope (CLSM, Olympus, Japan).

#### 2.3.4 Mature biofilm analysis and live/dead staining

A mature biofilm model was established using cell culture inserts (Irwin et al., 2023). *C. albicans* cell suspension (1 × 107 CFU/mL) was added to the inserts and incubated in RPMI-1640 for 48 h at 37 °C to form biofilms. At 24 h post-inoculation (to initiate biofilm formation), AbA (0.5 μg/mL) was added, and incubation was continued for an additional 24 h to reach mature biofilms. Biofilms were then stained using the LIVE/DEAD™ BacLight™ viability kit (Thermo Fisher Scientific, USA) according to the manufacturer's instructions. Z-stack images (step size: 1 μm) were acquired using a laser scanning confocal microscope (CLSM) with a 20 × objective. Biofilm thickness and the ratio of live/dead cells were quantified, and 3D reconstructions were generated using ImageJ software (NIH, USA) with the Bio-Formats plugin.

#### 2.3.5 Cytocompatibility assay of AbA

The L929 mouse fibroblast cell line was selected as the *in vitro* biocompatibility model. The cells were subcultured in high-glucose DMEM medium (Gibco, USA) supplemented with 10% fetal bovine serum (FBS; Hyclone, USA) and 1% penicillin-streptomycin double antibiotics (Thermo Fisher, USA) in an incubator at 37 °C with 5% CO_2_ and saturated humidity. When the cell confluency reached 80%-90%, the cells were digested with 0.25% trypsin-EDTA digestion solution (Hyclone, USA), centrifuged at 200 × g for 5 min, resuspended and counted. The cell density was adjusted to 8 × 10^3^ cells/mL. After the cells were cultured overnight for adherence, and two-fold serially diluted AbA solutions as well as 10 μL of methanol (served as the solvent control) were added to the corresponding wells. The cells were further cultured for 1, 2, and 3 days, respectively, to evaluate the effects of gradient concentrations of AbA and solvent methanol on the proliferation and viability of L929 mouse fibroblasts.

At the end of each culture period, the culture medium was aspirated, and 100 μL of pre-mixed CCK-8 working solution (containing 10% CCK-8 reagent and 90% serum-free medium, Beyotime, China) was added to each well. The plates were then incubated in the dark for 2 h. The absorbance value of each well was measured at 450 nm using a microplate reader, and the cytocompatibility of AbA toward L929 cells was assessed based on the measured absorbance values.

#### 2.3.6 RNA sequencing and analysis

Log-phase *C. albicans* cell suspension (1 × 108 CFU/mL) were treated with 1 × MIC AbA (0.0625 μg/mL) or sterile PBS (untreated control) for 12 h at 37 °C, with three independent biological replicates per group. Total RNA was extracted using TRIzol (Invitrogen, USA) reagent. RNA quality was then assessed using an Agilent 2100 Bioanalyzer, with samples having a RNA Integrity Number (RIN) ≥ 8.0 selected for subsequent library preparation. Library preparation and sequencing were performed on an Illumina NovaSeq platform. Quality-controlled reads were aligned to the *C. albicans* SC5314 reference genome. Differential gene expression analysis was performed using DESeq2 with thresholds set at |log2 fold change| > 1 and adjusted *p*-value < 0.05. Gene Ontology (GO) enrichment analysis was conducted using the clusterProfiler R package (v4.6.0) with adjusted *p*-value < 0.05 set as the significance threshold.

#### 2.3.7 Quantitative real-time PCR (qRT-PCR)

To validate RNA-seq results, total RNA was extracted from cells treated with AbA (0.0625, 0.25, 0.5 μg/mL) for 24 h. cDNA was synthesized using a PrimeScript RT reagent kit (TaKaRa, Japan) with gDNA Eraser (to further eliminate genomic DNA contamination). qRT-PCR was performed using SYBR Green Premix (Vazyme, China) on a QuantStudio system. The primers for oxidative stress-related genes (*TSA1, CAT1, NADPH* oxidase, *MCA1*) and the *C. albicans* reference gene GAPDH (GenBank ID: XM_712258) are listed in Supplementary Table S1. Relative gene expression levels were calculated using the 2^−ΔΔCT^ method, with GAPDH as the internal control.

#### 2.3.8 Analysis of cell apoptosis

The percentages of early and late apoptotic cells were determined using an Annexin V-FITC/PI apoptosis detection kit (Beyotime, China; Zhu et al., 2022). *C. albicans* cells were treated with AbA (0.0625–0.5 μg/mL) for 12 h, washed twice with PBS, and resuspended in the kit-supplied binding buffer. Cells were stained with Annexin V-FITC and PI for 15 min in the dark and analyzed immediately by flow cytometry (Beckman Coulter, USA). Data were analyzed using FlowJo software. Additionally, for the NAC intervention assay, *C. albicans* cells were first pretreated with the optimized concentration of NAC (10 mM) for 1 h, followed by co-incubation with 8 × MIC AbA for 12 h. After treatment, both the 8 × MIC AbA treated cells and the NAC + 8 × MIC AbA cells were subjected to the same washing, resuspension in binding buffer, and Annexin V-FITC/PI staining (15 min in the dark) as described above. These stained cells were then analyzed by flow cytometry immediately, and the percentages of Annexin V-FITC positive cells were quantified and compared using FlowJo software.

#### 2.3.9 Mitochondrial membrane potential (MMP) assay

MMP was assessed using the JC-1 fluorescent dye (Beyotime, China). AbA-treated log-phase *C. albicans* cells (1 × 106 CFU/mL, treated with 0.0625–0.5 μg/mL AbA for 12 h) were collected by centrifugation at 5,000 × g for 5 min at 4 °C, washed twice with PBS (pH 7.4), and incubated with JC-1 (5 μg/mL) at 37 °C in the dark for 30 min. After washing twice with PBS to remove excess dye, cells were analyzed by flow cytometry (Beckman Coulter CytoFLEX, USA) and confocal laser scanning microscopy (CLSM, Olympus FV3000, Japan). For CLSM imaging, each experimental group (Control, 1 × MIC, 4 × MIC, 8 × MIC AbA) was clearly labeled, with red fluorescence representing JC-1 aggregates (high MMP) and green fluorescence representing JC-1 monomers (low MMP).

### 2.3 TUNEL assay for DNA fragmentation

DNA fragmentation, a hallmark of late apoptosis, was detected using a TUNEL assay kit (Beyotime, China; Moore et al., 2021). Log-phase *C. albicans* cell suspension (1 × 106 CFU/mL) was treated with AbA (0.5 μg/mL) for 2 h, collected by centrifugation at 5,000 × g for 5 min at 4 °C, and fixed with 4% (w/v) paraformaldehyde at room temperature for 30 min. Cells were washed twice with PBS (pH 7.4), permeabilized with 0.1% Triton X-100 for 10 min, and then labeled with TUNEL reaction mixture at 37 °C in the dark for 60 min. Cells were visualized under a CLSM (Olympus FV3000, Japan), with TUNEL-positive cells (green fluorescence) counted in three random fields per sample. The positive rate was calculated, and three independent biological replicates were performed.

### 2.4 *In vivo* antifungal activity assessment

#### 2.4.1 Murine model of oral candidiasis

All animal procedures were approved by the Institutional Animal Care and Use Committee of University of Electronic Science and Technology of China. Six-to-eight-week-old female C57BL/6J mice (weight: 18–22 g, purchased from Chengdu Dossy Laboratory Animal Co., Ltd., China) were housed under specific pathogen-free (SPF) conditions with a 12-h light/dark cycle, *ad libitum* access to food and water, and acclimatized for 1 week before experimentation.

#### 2.4.2 Treatment groups and drug administration

All experimental procedures were performed in accordance with the Guide for the Care and Use of Laboratory Animals and approved by the local Ethics Committee [Approval No.: Lun Shen (Yan) 2024-662].

The mouse oral *C. albicans* model was established using the currently most commonly used intraoral swab method (Jungnickel and Jacobsen, 2022). To induce immunosuppression and facilitate fungal infection, cortisone acetate (220 mg/kg, dissolved in sterile normal saline, 0.1 mL/10 g body weight) was administered via subcutaneous injection on 3 days before infection, 1 day before infection, and 2 days after infection; meanwhile, 28.3 mg/mL tetracycline hydrochloride (Medchem Express, China) was added to the drinking water.

Pentobarbital sodium powder (Sigma, China) was accurately weighed and dissolved in sterile normal saline to prepare a 0.3% (w/v) solution. Anesthesia was induced via intraperitoneal injection at a dose of 50 mg/kg body weight. When the mice were anesthetized, a cotton ball was soaked in *C. albicans* cell suspension with a concentration of 1 × 10^8^ CFU/mL. The saturated cotton ball was evenly swabbed inside the mouse's oral cavity, then placed under the mouse's tongue, and removed after 2.5 h when the anesthetic effect subsided.

Starting at 24 h post-infection, local drug administration was conducted once daily for 3 consecutive days, following the specific procedure: First, mice were briefly anesthetized with isoflurane. Once anesthetized, 20 μL of the corresponding treatment solution (PBS or AbA at concentrations of 0.5 μg/mL) was aspirated with a pipette and slowly dispensed into the mouse's oral cavity—first evenly along the hard palate and bilateral buccal mucosa, followed by targeted dispensing onto the sublingual region. This ensured the treatment solution covered the entire surface of the oral mucosa. After dispensing, the mouse's head was kept slightly tilted upward for 10–15 s to allow the solution to fully infiltrate the mucosa without significant loss. The mice were then returned to their cages to recover from anesthesia, ensuring the efficacy of local administration. Administration on post-infection day 1, 2, and 3 (once daily) was completed before euthanasia on post-infection day 4, ensuring consistent treatment duration across all mice.

#### 2.4.3 Clinical scoring and fungal burden assessment

On post-infection day 4, mice were euthanized by intraperitoneal injection of an overdose of 0.3% pentobarbital sodium solution (150 mg/kg, approximately 0.5 mL/10 g body weight). Euthanasia was confirmed by observing the absence of chest movements for at least 2 min and the lack of pedal reflexes, in strict accordance with the guidelines of the Institutional Animal Care and Use Committee (IACUC) and the AVMA Guidelines for the Euthanasia of Animals. The tongues were then aseptically excised using sterile forceps and scissors.

For clinical scoring, tongues were photographed under standardized white light illumination between 9:00 a.m. and 11:00 a.m. The white plaque area was quantified using ImageJ software, and five independent investigators performed blind assessments using the following scoring system: Grade 0 (normal, white plaque area < 20%), Grade 1 (white plaque area 21%−50%), Grade 2 (white plaque area 51%−90%), and Grade 3 (white plaque area > 91%).

For fungal burden detection, each mouse's tongue was homogenized in 1 mL of PBS. After serial dilution of the homogenate, it was plated onto YPD agar medium supplemented with chloramphenicol. Following incubation at 37 °C for 48 h, colony-forming units (CFUs) were counted. The fungal burden was finally expressed as the number of CFUs per gram of tongue tissue.

#### 2.4.4 Histopathological analysis

Tongues were fixed in 10% neutral buffered formalin for 24 h, paraffin-embedded, and sectioned (7 μm thickness). Sections were stained with hematoxylin and eosin (H&E) to assess inflammation and tissue architecture, and with periodic acid-Schiff (PAS) to visualize fungal elements (hyphae and colonies). Stained sections were examined under a light microscope.

### 2.5 Statistical analysis

All *in vitro* experiments were performed with at least three independent biological replicates. Data are presented as mean ± standard error of the mean (S.E.M.). Statistical analysis was performed using GraphPad Prism 10. Comparisons between two groups were analyzed using an unpaired two-tailed Student's *t*-test. Comparisons among multiple groups were analyzed by one-way analysis of variance (ANOVA) followed by Dunnett's *post hoc* test (compared to control) or Tukey's *post hoc* test (for all pairwise comparisons). A *p*-value of less than 0.05 was considered statistically significant.

## 3 Result

### 3.1 AbA exhibits potent concentration- and time-dependent antifungal activity

**Figure 1:**
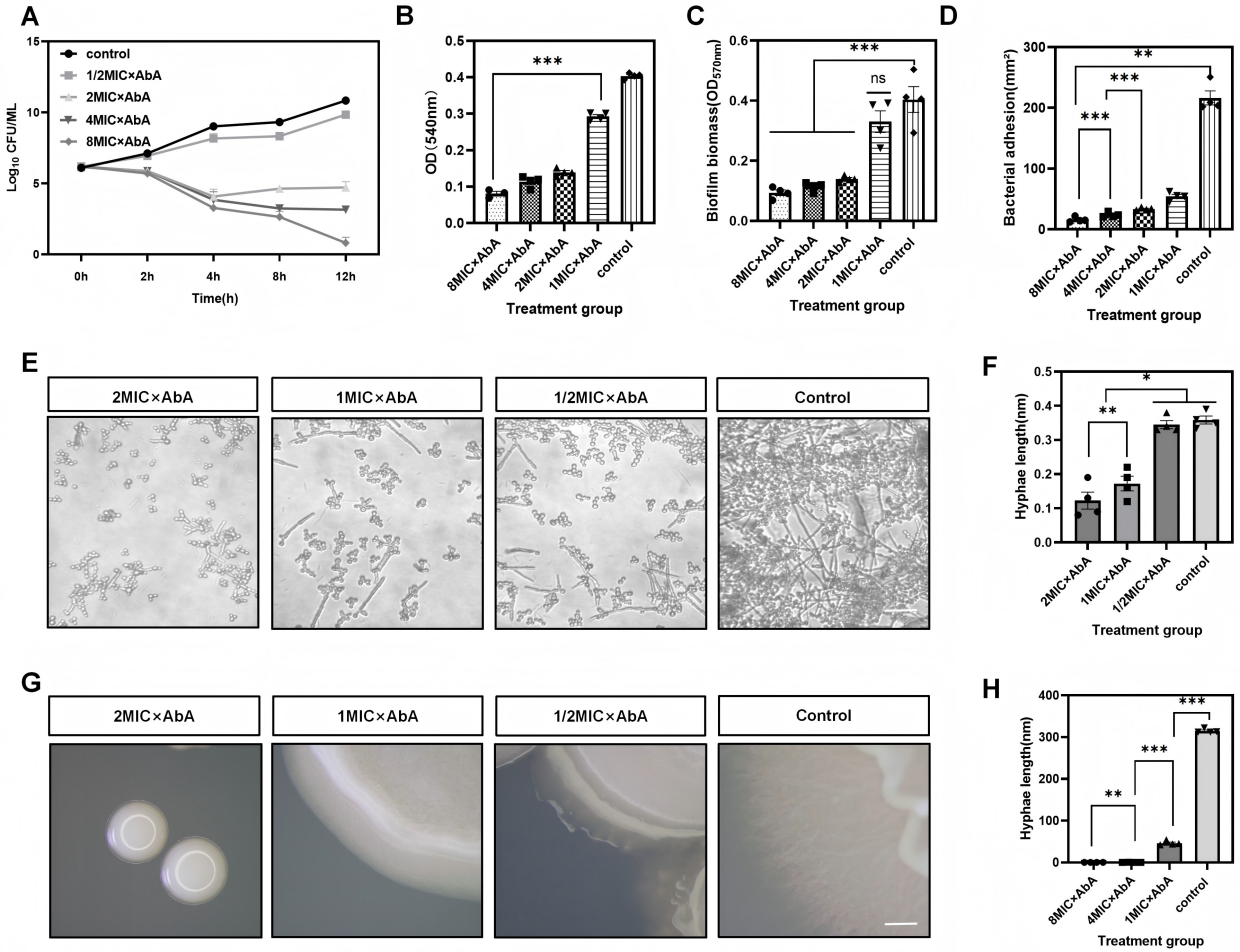
AbA inhibits growth and virulence factors of *C. albicans* SC5314. **(A)** Time-kill curves of *C. albican*s treated with AbA at concentrations of 0.03125 (1/2 × MIC), 0.125 (2 × MIC), 0.25 (4 × MIC), and 0.5 (8 × MIC) μg/mL over 12 h. Data are presented as mean ± SD (*n* = 3 independent biological replicates). **(B)** Quantitative analysis of early biofilm formation measured by crystal violet staining. Biofilms were treated with the indicated concentrations of AbA for 2 h. **(C)** Effect of AbA on the production of EPS, as determined by the phenol-sulfuric acid method. **(D)** Adhesion capability of *C. albicans* to L929 mammalian cell monolayers following AbA treatment. Adhesion area was quantified using ImageJ. **(E, F)** Representative micrographs **(E)** and quantitative analysis **(F)** of hyphal formation in Spider liquid medium after 24 h treatment with AbA. Hyphal length was measured using Image J. Scale bar = 50 μm. **(G, H)** Representative images of colony morphology **(G)** and hyphal formation **(H)** on Spider solid agar plates after 4 days of incubation with AbA. Data are presented as mean ± SD from four independent experiments. Statistical significance was determined by one-way ANOVA with Dunnett's *post hoc* test (ns: *p* > 0.05 (not significant), *p < 0.05, **p < 0.01, ***p < 0.001.).

The MIC of AbA against *C. albicans* SC5314 was determined to be 0.0625 μg/mL. Time-kill kinetics assays demonstrated the potent fungicidal activity of AbA (Figure 1A). Treatment with concentrations ≥ 2 × MIC (0.125 μg/mL) resulted in a progressive, time-dependent reduction in colony-forming units (CFUs), with 4 × MIC and 8 × MIC achieving near-complete eradication within 12 h.

### 3.2 AbA suppresses key virulence factors of *C. albicans*

AbA significantly impaired biofilm-associated pathogenicity. Crystal violet staining revealed a dose-dependent inhibition of early biofilm formation at concentrations as low as 2 × MIC (Figure 1B). This was accompanied by a concomitant reduction in the production of extracellular polymeric substances (EPS; Figure 1C), which are critical for biofilm matrix integrity. Furthermore, AbA treatment markedly attenuated the adhesion capacity of *C. albicans* to host L929 cells, reducing its ability to initiate infection (Figure 1D).

The morphological transition from yeast to hyphae, a cornerstone of *C. albicans* virulence, was profoundly inhibited by AbA. In Spider liquid medium, AbA treatment caused a concentration-dependent reduction in hyphal formation. Quantitative analysis confirmed a significant decrease in hyphal length compared to the control (Figures 1E, F). This anti-hyphal effect was consistently observed on solid Spider medium, where AbA-treated colonies exhibited a predominantly yeast-form morphology, in stark contrast to the extensive hyphal outgrowth in the control (Figures 1G, H).

### 3.3 AbA compromises cell membrane integrity and cellular ultrastructure

**Figure 2:**
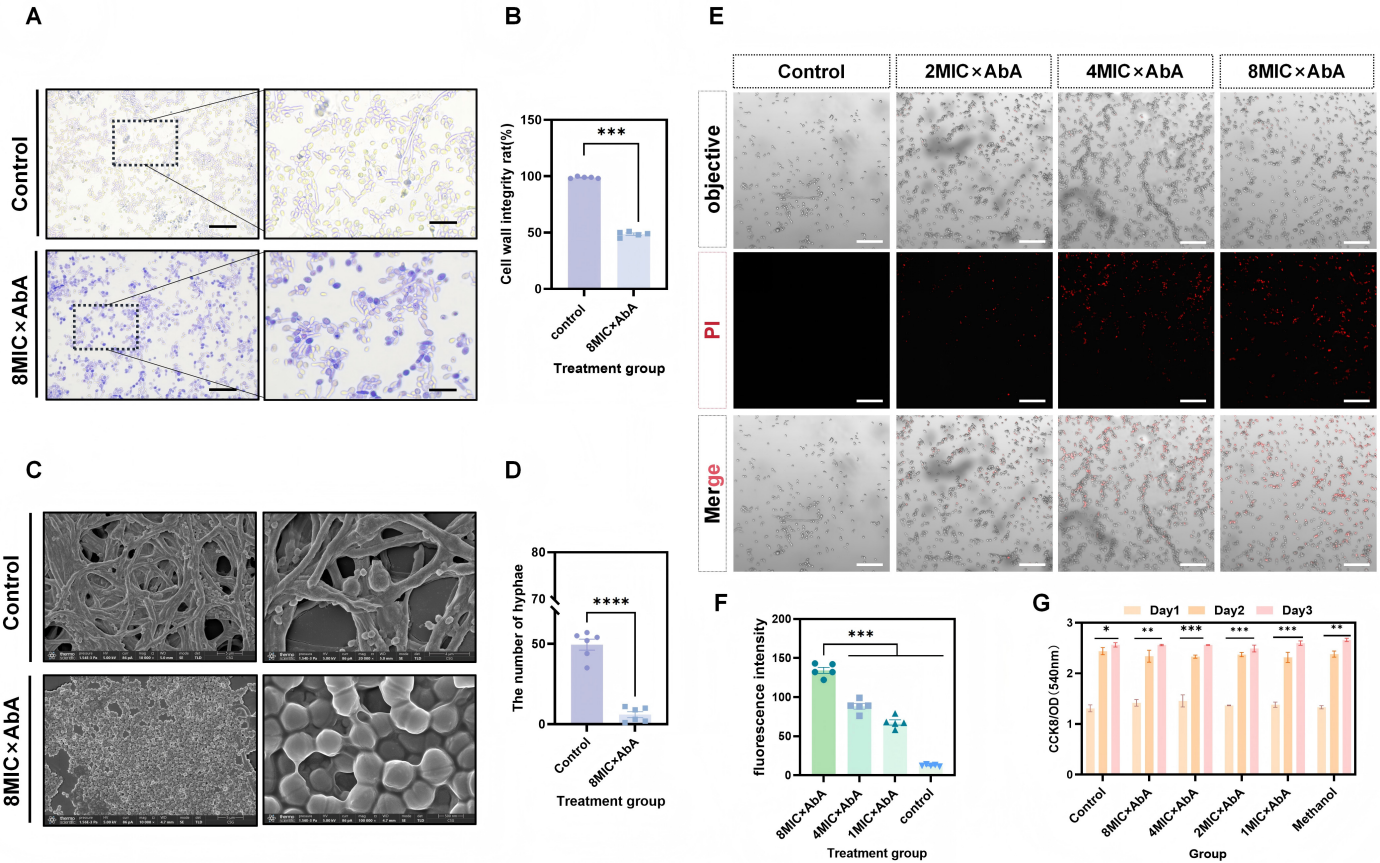
AbA compromises cellular integrity and ultrastructure without mammalian cell toxicity. **(A, B)** Assessment of cell wall integrity by crystal violet uptake assay. *C. albicans* cells were treated with 0.5 μg/mL (8 × MIC) AbA for 10 h. **(A)** Representative micrographs; scale bar = 50 μm. **(B)** Quantitative analysis of cells with compromised integrity. **(C, D)** Scanning electron microscopy (SEM) images revealing the ultrastructural alterations in *C. albicans* after treatment with 0.5 μg/mL (8 × MIC) AbA. **(C)** Representative images show surface wrinkling and collapse of AbA-treated cells compared to the smooth surface of untreated control cells. Magnification: 20,000× . **(D)** Quantitative analysis of hyphal formation. **(E, F)** Evaluation of membrane integrity by propidium iodide (PI) staining. **(E)** Representative fluorescence micrographs of cells treated with the indicated concentrations of AbA (0.0625–0.5 μg/mL). PI (red) stains nuclei of cells with permeabilized membranes. Scale bar = 100 μm. **(F)** Quantitative analysis of PI fluorescence intensity measured by a microplate reader. **(G)** Cytocompatibility of AbA on mouse L929 fibroblast cells after 48 h of treatment, as assessed by CCK-8 assay. Data are normalized to the untreated control (100% viability). Data are presented as mean ± SD from five independent experiments. Statistical significance was determined by one-way ANOVA with Dunnett's *post hoc* test (*p < 0.05, **p < 0.01, ***p < 0.001, ****p < 0.0001).

Crystal violet uptake assays indicated that treatment with 8 × MIC AbA (0.5 μg/mL) significantly increased dye absorption, suggesting enhanced membrane permeability and compromised cell wall integrity (Figures 2A, B). Scanning electron microscopy (SEM) provided visual evidence of ultrastructural damage. AbA-treated cells displayed extensive surface wrinkling, collapse, and a near-complete absence of hyphal structures, contrasting with the smooth, intact surface of untreated control cells (Figures 2C, D). This membrane damage was further confirmed by PI staining. As the concentration of AbA increased, so did the intensity of PI fluorescence, indicating a dose-dependent loss of membrane integrity (Figures 2E, F). Importantly, the cytocompatibility assay results showed that AbA had no significant cytotoxic effects on mammalian L929 fibroblast cells at the tested concentrations. Statistical comparison between the solvent control group (0.5% methanol) and the blank control group confirmed that the solvent had no significant impact on cell viability (Figure 2G), indicating the selective antifungal action of AbA.

### 3.4 . AbA induces oxidative stress and is associated with apoptotic cell death in *C. albicans*

**Figure 3:**
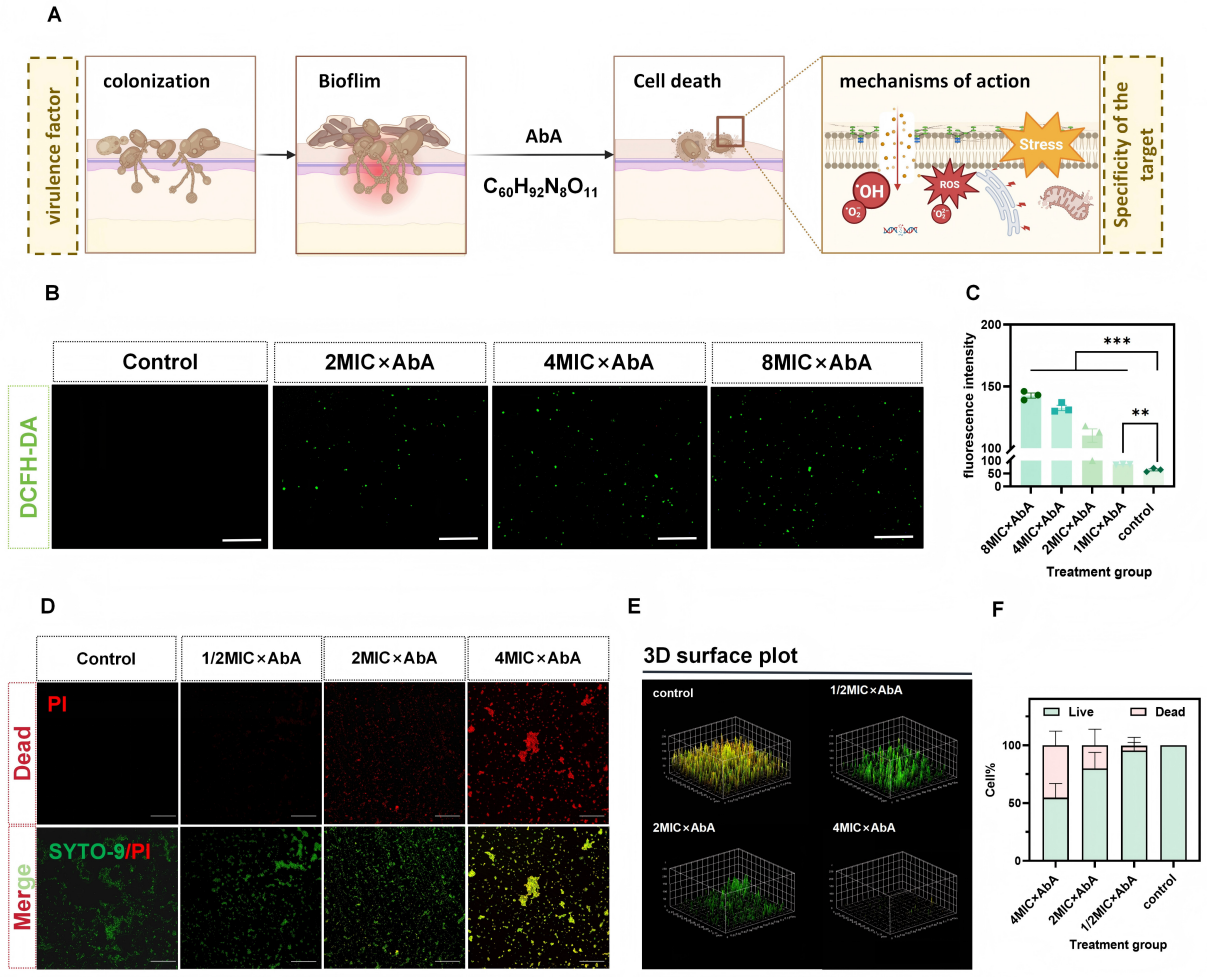
AbA induces oxidative stress and triggers apoptosis in *C. albicans* biofilms. **(A)** Proposed model for the antifungal mechanism of AbA, involving membrane targeting, ROS accumulation, and induction of the mitochondrial apoptotic pathway. **(B, C)** Intracellular ROS detection via DCFH-DA fluorescence. **(B)** Representative confocal micrographs of *C. albicans* biofilms after 4 h treatment with 2 × MIC, 4 × MIC, 8 × MIC AbA (or PBS as Control); scale bar = 100 μm. **(C)** Quantitative analysis of DCFH-DA fluorescence intensity (reflecting intracellular ROS levels) in the groups shown in **(B)**; data are presented as mean ± SD (*n* = 3 independent biological replicates). **(D, E)** AbA-induced cell death in mature *C. albicans* biofilms. **(D)** LIVE/DEAD staining results (Control, 1/2 × MIC, 2 × MIC, 4 × MIC AbA groups): PI (red, dead cells), SYTO-9 (green, live cells), and merged images; scale bar = 50 μm. **(E)** Representative 3D reconstructions (from z-stack confocal images) of *C. albicans* biofilms corresponding to the groups in **(D)**. **(F)** Quantitative analysis of live/dead cell proportions in C. albicans biofilms across the groups shown in **(D)**; data are presented as mean ± SD (*n* = 3 independent biological replicates). Statistical significance was determined by one-way ANOVA with Dunnett's *post hoc* test (**p < 0.01, ***p < 0.001).

We hypothesized that the membrane damage induced by AbA could lead to intracellular ROS accumulation, triggering apoptosis (Figure 3A). DCFH-DA staining confirmed a significant, dose-dependent increase in intracellular ROS levels following AbA treatment (Figures 3B, C). We next investigated if this ROS burst could induce apoptosis within mature biofilms, a treatment-recalcitrant state. Confocal microscopy analysis of LIVE/DEAD-stained biofilms showed that 8 × MIC AbA treatment drastically reduced biofilm viability and thickness (3D reconstruction). The ratio of dead (red) to live (green) cells increased significantly, demonstrating potent antibiofilm activity (Figures 3D–F).

### 3.5 Transcriptomic and molecular analysis confirms apoptosis activation

**Figure 4:**
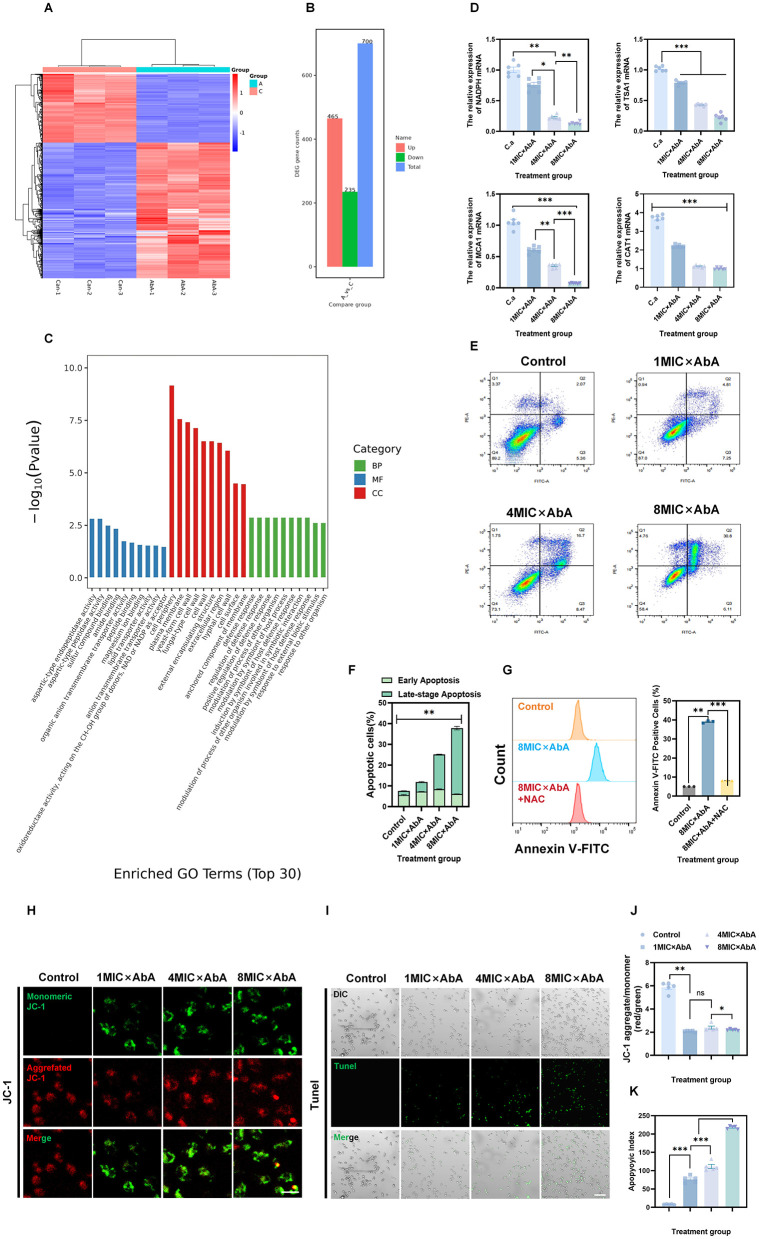
Transcriptomic and functional validation of AbA-induced mitochondrial apoptosis in *C. albicans*. **(A–C)** After treatment with AbA at 1 × MIC (0.0625 μg/mL) for 12 h, RNA-sequencing (RNA-seq) analysis was performed on *C. albicans*. **(A)** Volcano plot of differentially expressed genes (DEGs; screening criteria: |log_2_ fold change| > 1, adjusted *p*-value < 0.05); **(B)** Bar chart of differentially expressed genes (statistical counts of upregulated and downregulated DEGs: 465 upregulated and 235 downregulated, with the same screening criteria as in **A**). **(C)** Gene Ontology (GO) enrichment analysis of biological processes for the downregulated DEGs, and revealing the oxidative stress response pathway was significantly downregulated. **(D)** qRT-PCR validation of key oxidative stress-related genes (*TSA1, NADPH oxidase, MCA1, CAT1*) in cells treated with AbA (0.0625, 0.25, 0.5 μg/mL) for 24 h. data are presented as mean ± SD (*n* = 6 independent biological replicates). **(E, F)** Quantification of apoptosis by Annexin V-FITC/PI staining and flow cytometry. data are presented as mean ± SD (*n* = 3 independent biological replicates). **(G)** Effect of NAC pretreatment on AbA-induced apoptosis. Quantitative analysis of Annexin V-FITC/PI positive cells in Control, 8MIC × AbA, and 8MIC × AbA+NAC groups. data are presented as mean ± SD (*n* = 3 independent biological replicates). **(H, J)** Assessment of mitochondrial membrane potential (MMP) via JC-1 staining. **(H)** Representative fluorescence micrographs (red: JC-1 aggregates, high MMP; green: JC-1 monomers, low MMP); scale bar = 5 μm. **(J)** Quantitative analysis of the red/green fluorescence intensity ratio in Control, 1MIC × AbA, 4MIC × AbA, and 8MIC × AbA groups. data are presented as mean ± SD (*n* = 5 independent biological replicates). **(I, K)** Detection of DNA fragmentation via TUNEL assay (late apoptosis hallmark). **(I)** Representative fluorescence micrographs of TUNEL-positive cells (green) after 0.5 μg/mL AbA treatment for 2 h; scale bar = 50 μm. **(K)** Quantitative analysis of TUNEL-positive cells. data are presented as mean ± SD (*n* = 5 independent biological replicates). Statistical significance was determined by one-way ANOVA with Dunnett's *post hoc* test (ns: *p* > 0.05 (not significant), *p < 0.05, **p < 0.01, ***p < 0.001).

RNA-seq analysis of AbA-treated cells revealed significant transcriptional reprogramming (Figure 4A). Gene Ontology (GO) enrichment analysis of differentially expressed genes (DEGs)—which were identified via DESeq2 analysis to total 700 significantly dysregulated genes, including 465 upregulated genes (log_2_FC > 1) and 235 downregulated genes (log_2_FC < −1), with adjusted *p* < 0.05 (Figure 4B), GO enrichment analysis showed a significant downregulation of processes related to oxidative stress response (Figure 4C).

qRT-PCR validation confirmed the dose-dependent downregulation of key oxidative stress defense genes, MCA1 (GenBank ID: XM_711927) was selected as a candidate gene for qRT-PCR validation based on its well-documented role in fungal apoptosis, rather than being identified as a DEG in RNA-seq. including *TSA1* (thiol-specific antioxidant), CAT1 (catalase), a *NADPH oxidase* gene (Figure 4D). This downregulation of the antioxidant defense system presumably exacerbates ROS-induced cellular damage.

Pretreatment of *C. albicans* with NAC, a ROS-specific scavenger, completely reversed AbA-induced intracellular ROS elevation to the control level (Supplementary Figure S1). Furthermore, flow cytometric analysis confirmed that NAC pretreatment abrogated the AbA-induced increase in apoptotic cell populations (Figure 4G), underscoring ROS as a critical upstream mediator of AbA-triggered apoptosis. Flow cytometry analysis using Annexin V-FITC/PI staining provided direct evidence of apoptosis. AbA treatment led to a significant, concentration-dependent increase in the population of early (Annexin V+/PI-) and late (Annexin V+/PI+) apoptotic cells (Figures 4E, F). Loss of mitochondrial membrane potential (MMP), a key event in intrinsic apoptosis, was detected using JC-1 staining. AbA-treated cells showed a pronounced shift from red (aggregated JC-1, high MMP) to green (monomeric JC-1, low MMP) fluorescence, indicating MMP collapse (Figures 4H, J). To confirm that MMP loss is ROS-dependent, we further evaluated the effect of NAC pretreatment on MMP: consistent with ROS scavenging activity, NAC significantly reversed the AbA-induced shift toward JC-1 monomeric form, restoring the proportion of aggregated JC-1-positive cells to the control level (Supplementary Figure S2). This result confirms that ROS accumulation is a prerequisite for AbA-induced mitochondrial dysfunction.

Finally, TUNEL assay confirmed the occurrence of DNA fragmentation—the terminal stage of apoptosis—in AbA-treated cells (Figures 4I, K). Complementary experiments showed that NAC pretreatment markedly reduced the number of TUNEL-positive cells in AbA-treated cultures (Supplementary Figure S3), directly linking ROS accumulation to downstream DNA damage and validating the integrity of the “ROS to mitochondrial dysfunction to DNA fragmentation” signaling cascade.

To distinguish between cell apoptosis and non-specific necrosis, we performed LDH release assay (Supplementary Figure S4). The results showed that the LDH release rate in the AbA group was not significantly different from that in the control group. Since LDH release is a hallmark of cell necrosis but not apoptosis, this finding confirms that AbA-induced cell death occurs via apoptosis.

### 3.6 Topical AbA application ameliorates oral candidiasis *in vivo*

**Figure 5:**
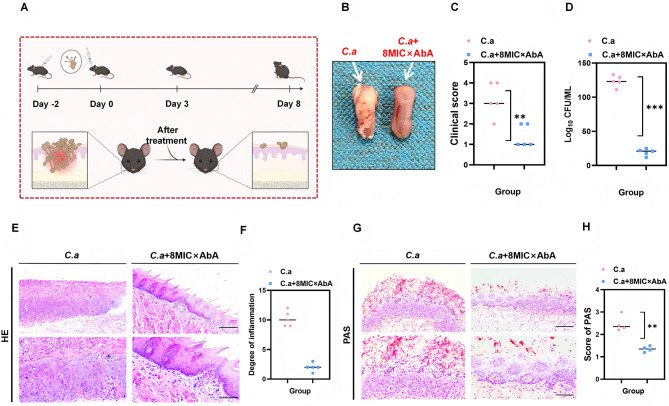
Topical application of AbA ameliorates oral candidiasis in a murine model. **(A)** Experimental workflow of the oral candidiasis model and treatment regimen. C57BL/6J mice were immunosuppressed with cortisone acetate, sublingually infected with *C. albicans* (1 × 108 CFU/mL), and treated with AbA or PBS (vehicle) for 3 consecutive days post-infection. **(B)** Representative macroscopic images of mouse tongues from the PBS-treated (vehicle) and AbA-treated (8MIC × AbA) groups at the experimental endpoint. **(C)** Clinical scoring of oral lesion severity (0–3 scale) based on the area of white plaques. Each data point represents the score of one mouse, as assessed by five independent investigators via blinded assessments. **(D)** Fungal burden in tongue tissue, quantified by plating tissue homogenates and enumerating CFUs. Data are presented as CFUs per gram of tongue tissue. The dashed line indicates the limit of detection. **(E, F)** Histopathological analysis of tongue sections via H&E staining. Scale bars = 100 μm. **(G, H)** Assessment of fungal burden and morphology in tongue sections via PAS staining. Scale bars = 100 μm. Data in **(C, D, F, H)** are presented as mean ± SD (*n* = 5 mice per group). Statistical significance was determined by unpaired two-tailed Student's *t*-test (**p < 0.01, ***p < 0.001).

The therapeutic efficacy of AbA was evaluated in a murine model of oral candidiasis (Figure 5A). Macroscopic examination showed that vehicle-treated mice developed extensive white plaques and significant tongue edema. In contrast, mice treated with topical AbA exhibited markedly reduced plaque areas and minimal signs of inflammation (Figure 5B). This was corroborated by a significantly lower clinical score in the AbA group (Figure 5C). Quantitative culture of tongue homogenates revealed a substantial reduction (*p* < 0.01) in the fungal burden (CFU/g of tissue) in AbA-treated mice compared to the vehicle control (Figure 5D). Histopathological analysis further supported these findings. H&E staining showed that AbA treatment reduced epithelial hyperplasia and inflammatory cell infiltration (Figures 5E, F). PAS staining clearly demonstrated that AbA treatment significantly inhibited fungal colonization and hyphal penetration of the mucosal tissue (Figures 5G, H).

## 4 Discussion

The escalating prevalence of azole-resistant *C. albicans* strains highlights an urgent need for antifungal agents with novel mechanisms of action. While AbA is well-documented to inhibit inositol phosphorylceramide (IPC) synthase, the present study suggests a potential secondary mechanism involving the participation of ROS in mitochondrial apoptosis, rather than definitively demonstrating ROS-mediated induction of apoptosis. This work significantly advances our understanding of AbA's polypharmacological properties and its potential as both an anti-virulence and pro-apoptotic agent, particularly for the treatment of oral candidiasis.

As the rate-limiting enzyme in fungal sphingolipid biosynthesis, IPC synthase is critical for maintaining the lipid composition, fluidity, and structural integrity of fungal cell membranes (Usmani et al., 2023; Cahoon et al., 2024). By inhibiting IPC synthase, AbA initially disrupts sphingolipid homeostasis. This disruption not only impairs the structural foundation of fungal cell membranes but also interferes with the function of membrane-localized transporters—such as *CDR1* and *CDR2*, the major efflux pumps mediating azole resistance—and signaling complexes (Czajka et al., 2023).

This membrane perturbation creates two favorable conditions for activating the secondary apoptotic pathway (Ponde et al., 2021; Shaito et al., 2022). First, it increases membrane permeability to AbA itself, facilitating intracellular drug accumulation and preventing the selection of drug-resistant mutants caused by “insufficient drug concentrations.” Second, it diminishes the membrane's capacity to scavenge ROS and compromises the stability of the outer mitochondrial membrane, rendering ROS accumulation and subsequent mitochondrial dysfunction more likely to occur.

Subsequently, the AbA-triggered ROS burst further amplifies cellular damage. Oxidative stress not only directly oxidizes DNA, proteins, and lipids but also downregulates the expression of antioxidant genes (e.g., *TSA1, CAT1*), forming a “ROS amplification loop” that accelerates mitochondrial depolarization and apoptosis (Liu et al., 2022; Ulasov et al., 2022; Sazykin and Sazykina, 2023). By targeting two genetically and functionally independent pathways, this dual mechanism renders fungi less likely to develop resistance through a single mutational event.

Our findings confirm that AbA exerts potent, concentration-dependent fungicidal effects against *C. albicans* (Figure 1A). More importantly, we demonstrate that AbA effectively disrupts key virulence-associated pathways. It significantly inhibits hyphal formation (Figures 1E–H)—a critical determinant of tissue invasion and pathogenicity in *C. albicans*. Notably, the inhibition of hyphal formation may be associated with the downregulation of key regulatory genes such as *HWP1, ALS1*, and *EFG1*, which are essential for hyphal development and adhesion in *C. albicans*; this hypothesis warrants further verification via RT-PCR and Western blot assays in subsequent studies. Additionally, it impairs biofilm formation and adhesion (Figures 1B–D), traits essential for establishing persistent infections and mediating antimicrobial resistance. This multi-faceted targeting of virulence factors suggests that AbA may not only eradicate *C. albicans* but also prevent the establishment of hard-to-treat infections.

The central novel finding of this study is elucidation of a potential apoptotic pathway involving AbA, with ROS playing a participatory role. We provide a robust evidence chain supporting ROS participation: (1) AbA induces membrane damage (Figures 2A–F); (2) this membrane damage leads to a surge in intracellular ROS levels (Figures 3B, C); (3) transcriptomic and qRT-PCR analyses reveal downregulation of key antioxidant genes (*TSA1, CAT1*), which further exacerbates oxidative stress (Figures 4A–D); (4) ROS overload triggers mitochondrial dysfunction, as evidenced by the collapse of mitochondrial membrane potential (MMP; Figures 4E, F); (5) this dysfunction initiates apoptosis, confirmed by the down-regulation of *MCA1* (the key cysteine protease of *C. albicans* intrinsic apoptosis pathway), phosphatidylserine externalization (Annexin V-FITC/PI staining, Figures 4H, J) and DNA fragmentation (TUNEL assay, Figures 4I, K). Notably, this mechanism is particularly effective against biofilms (Figures 3D–F)—a major challenge in clinical antifungal therapy.

To further clarify the causal hierarchy of the aforementioned apoptotic pathway, we conducted targeted intervention experiments for verification: Results from N-acetylcysteine (NAC)-mediated ROS scavenging assays clearly highlight the participatory role of ROS in AbA-induced apoptosis of *C. albicans*. These findings confirm the regulatory effect of NAC on ROS levels and apoptosis rates (Supplementary Figure S1 and Figure 4G) and suggest a potential regulatory hierarchy within the pathway—ROS accumulation may act as a potential upstream signal that contributes to AbA-triggered cell death. This is consistent with the classical understanding of fungal apoptosis: oxidative stress is a conserved driver of apoptotic signaling in fungi, and excessive ROS disrupts cellular redox homeostasis, thereby initiating downstream apoptotic cascades. Importantly, these results do not rule out the possibility that ROS elevation and apoptosis may interact in a bidirectional manner, nor do they exclude the involvement of other parallel pathways that may contribute to apoptosis. Thus, we cannot definitively conclude that ROS is the “cause” of apoptosis; instead, ROS is likely a key mediator involved in the progression of AbA-induced apoptotic events.

As a core event in the intrinsic apoptotic pathway, MMP collapse serves as a critical intermediate node connecting ROS accumulation and DNA fragmentation (Supplementary Figure S2). Building on the above finding that ROS is an upstream signal, NAC's ability to restore AbA-impaired MMP indicates that ROS-mediated mitochondrial dysfunction is not random cellular damage but a targeted regulatory step in AbA-induced apoptosis. It is well-established that mitochondria play dual roles in ROS metabolism: they are both the primary source of intracellular ROS and the core target of ROS-induced damage (Huangteerakul et al., 2021; Andrieux et al., 2021). Excessive ROS generated by AbA-induced oxidative stress can oxidize lipids and proteins in the mitochondrial membrane, directly leading to MMP loss; this loss, in turn, disrupts mitochondrial homeostasis and promotes the release of pro-apoptotic factors into the cytoplasm. Our results suggest that AbA may exploit this interaction to form a positive feedback loop: AbA initially induces oxidative stress and ROS accumulation, which damages mitochondria and causes MMP collapse; the damaged mitochondria then produce more ROS, amplifying the apoptotic signal. This mitochondrial ROS amplification loop can well explain the concentration-dependent apoptotic effect of AbA observed in flow cytometry assays (Figures 4E, F)—higher AbA concentrations further disrupt cellular redox balance, exacerbate mitochondrial damage, and thus enhance the ROS amplification effect, ultimately leading to a more pronounced apoptotic response.

Moving to the terminal stage of apoptosis, we found that NAC significantly inhibits AbA-induced DNA fragmentation (Supplementary Figure S3)—a finding that further supports the downstream regulatory role of ROS signaling in the apoptotic execution phase. DNA fragmentation is a hallmark of late apoptosis, tightly regulated by caspase-like proteases and endonucleases in fungi (Bhatt et al., 2023). Our data indicate that ROS accumulation is a prerequisite for activating these apoptotic effector molecules: by scavenging ROS, NAC blocks upstream signal transduction leading to DNA damage, thereby inhibiting the completion of apoptotic execution. In the context of existing antifungal research, ROS-dependent DNA damage has been linked to the cytotoxicity of azoles and echinocandins. However, AbA is unique in that it specifically targets the oxidative stress defense system—by downregulating key antioxidant genes such as *TSA1* and *CAT1*, AbA impairs the cell's ability to tolerate ROS accumulation, suggesting a distinct mechanism for amplifying ROS-induced DNA damage.

The LDH release assay (Supplementary Figure S4) provides key evidence for clarifying the type of cell death induced by AbA. No significant LDH release was observed in the AbA-treated group—a core distinguishing feature between apoptosis and necrosis. This finding holds important clinical value: apoptotic cell death is generally less immunogenic than necrosis, whereas membrane rupture associated with necrosis releases pro-inflammatory cellular components, triggering host inflammatory responses that may exacerbate tissue damage during fungal infections. By eliminating C. albicans through inducing apoptosis rather than necrosis, AbA may possess a unique therapeutic advantage, a characteristic that warrants further validation in *in vivo* models.

The *in vivo* efficacy of topical AbA administration was highly promising in an immunocompetent mouse model (Figure 5). The significant reductions in fungal burden and pathological damage highlight its therapeutic potential for topical applications, such as the treatment of oral thrush. Although a high fungal inoculum was used in the model to ensure robust infection, the clear therapeutic effect demonstrates AbA's ability to act in a complex biological microenvironment.

Despite these promising findings, the present study has several limitations. First, only the *C. albicans* SC5314 strain was used, limiting the generalizability of our results to other clinical isolates. Second, the *in vivo* model employed high inoculum doses and immunosuppressive conditions, which may not fully recapitulate clinical scenarios. Third, the systemic pharmacokinetics and toxicity profile of AbA remain unknown. Furthermore, while topical AbA was effective in the mouse oral candidiasis model, caution is warranted for clinical translation, and additional long-term assessments of oral mucosal toxicity are required. Fourth, while pharmacological inhibition points to a critical role for ROS, future studies employing genetic tools to manipulate ROS generation or scavenging in a temporally controlled manner would help to further delineate the causal hierarchy within the proposed apoptotic pathway.

In summary, the present study clearly delineates the complete pathway of AbA-induced apoptosis in *C. albicans*: AbA first downregulates the oxidative stress defense system, leading to ROS accumulation; ROS then mediates mitochondrial membrane potential collapse, activates downstream apoptotic effector molecules, and ultimately induces DNA fragmentation. Of key importance is the synergistic effect between this newly discovered apoptotic mechanism and AbA's established role as an IPC synthase inhibitor. Based on this, we propose a synergistic model: AbA-mediated inhibition of sphingolipid synthesis may disrupt cell membrane structure from the initial stage, promoting the initial uptake of AbA by cells on the one hand and reducing membrane stability on the other. This primary membrane damage may lay the foundation for the subsequent explosive accumulation of ROS and apoptotic cell death. This dual mechanism of action may explain AbA's significant antifungal activity and its potential to inhibit the development of drug resistance.

In conclusion, beyond its well-characterized inhibition of sphingolipid synthesis, AbA executes a sophisticated antifungal strategy by targeting essential cellular processes and triggering programmed cell death in *C. albicans*. Through its ability to induce ROS-mediated apoptosis and suppress virulence traits, AbA represents a promising multi-target therapeutic candidate for both oral and invasive drug-resistant fungal infections. This study provides a robust foundation for the further development of AbA and underscores the induction of fungal apoptosis as a viable strategy for combating drug-resistant fungal infections.

## Data Availability

The raw data supporting the conclusions of this article will be made available by the authors, without undue reservation.
