# Supplementary material for: ROS-implicated apoptosis in Candida albicans: mechanistic insights into Aureobasidin A's antifungal activity
Source: Front Microbiol. 2026 Feb 6;17:1725921. doi: 10.3389/fmicb.2026.1725921 (PMC12920453; doi:10.3389/fmicb.2026.1725921)
Supplement: Supplementary file 1 [file Table_1.docx]

Supplementary Material

# Supplementary Data

**1.1 Experimental section**

**1.1.1 Rescue Experiment of AbA-Induced Intracellular ROS Accumulation in *C. albicans***

*Candida albicans (C. albicans)* SC5314 was cultured overnight at 37°C with shaking at 200 rpm in YPD liquid medium until the logarithmic growth phase. Cells were harvested by centrifugation at 3000 × g for 5 min at 4°C, washed twice with sterile PBS (pH 7.4), resuspended, and adjusted to a final concentration of 1×10⁶ CFU/mL. The cell suspension was divided into three groups with triplicate wells each, and treated as follows: 1) Control group: only an equal volume of sterile PBS (pH 7.4) was added; 2) AbA-only group (AbA alone): AbA was added to a final concentration of 8×MIC (0.5 μg/mL, equivalent to 8×MIC), followed by incubation at 37°C with shaking for 4 h; 3) Experimental group (NAC+AbA): cells were first pretreated with 10 mM N-acetylcysteine (NAC) for 2 h, then AbA was added to a final concentration of 8×MIC (0.5 μg/mL), and incubation was continued at 37°C with shaking for 4 h (the total treatment time was consistent with that of the AbA-only group). After treatment, cells in each group were collected by centrifugation at 3000 × g for 5 min at 4°C, washed twice with PBS (pH 7.4), and incubated with 10 μM DCFH-DA at 37°C in the dark with gentle shaking for 30 min. The cells were washed again to remove excess probe. The cells were washed again to remove excess probe. Subsequently, the cells were resuspended in an appropriate volume of sterile PBS, and the fluorescence intensity was measured using a microplate reader (excitation wavelength: 488 nm; emission wavelength: 525 nm).

**1.1.2 NAC-Mediated Protection Experiment of AbA-Induced Mitochondrial Dysfunction and Apoptosis in *C. albicans***

Strain culture and pretreatment were performed as described in Section 1.1.1. The cell suspension was divided into three groups (with triplicate wells each for parallel imaging and quantification), and the treatments were as follows: 1) Control group: only an equal volume of sterile PBS was added, followed by incubation at 37°C with shaking at 200 rpm for 14 h (total treatment time = CsA pretreatment time+AbA incubation time, consistent with the experimental group); 2) AbA-only group: AbA was added to a final concentration of 8×MIC (0.5 μg/mL), and incubated at 37°C with shaking for 12 h; 3) Experimental group (CsA+AbA): cells were first pretreated with 5 μM cyclosporin A (CsA) for 2 h, then AbA was directly added to a final concentration of 8×MIC without medium replacement (to avoid disturbing cell status), and incubation was continued at 37°C with shaking for 12 h (total treatment time of 14 h).

After the respective treatments, cells were collected by centrifugation at 5,000 × g for 5 min at 4°C, washed twice with sterile PBS (pH 7.4), and resuspended in 500 μL PBS. JC-1 staining solution was added to a final concentration of 5 μg/mL, and the cells were incubated at 37°C in the dark for 30 min. Following incubation, cells were washed three times with PBS to remove unbound free dye and avoid background fluorescence interference. A 100 μL aliquot of the stained cell suspension was dropped onto confocal-specific culture dishes, and images were acquired using a confocal laser scanning microscope (CLSM, Olympus FV3000, Japan).

Cells were treated as described above, collected by centrifugation at 5,000 × g for 5 min at 4°C, and fixed with 4% (w/v) paraformaldehyde at room temperature for 30 min. After being washed twice with PBS (pH 7.4), cells were permeabilized with 0.1% Triton X-100 for 10 min, then incubated with TUNEL reaction mixture at 37°C in the dark for 60 min. Cells were visualized under a confocal laser scanning microscope (CLSM, Olympus FV3000, Japan), with TUNEL-positive cells (green fluorescence) counted in three random fields per sample. The positive rate was calculated, and three independent biological replicates were performed.

**1.1.3 LDH Release Assay for Quantification of Cell Death**

Additionally, LDH release assay was conducted to distinguish between the cell necrosis and apoptosis, with grouping and procedures as follows: Cells were prepared as described above and seeded into 96-well plates (100 μL per well, 1×10⁶ cells/mL). Five groups were established with triplicate wells per group: 1) Control group (sterile PBS only); 2) 1×MIC AbA group; 3) 4×MIC AbA group; 4) 8×MIC AbA group; 5) 5 mM hydrogen peroxide (H_2_O_2_) group. All groups were incubated at 37°C with shaking at 200 rpm for 4 h. After incubation, the plate was centrifuged at 5,000 × g for 5 min at 4°C. Fifty microliters of supernatant from each well was transferred to a new 96-well plate, followed by the addition of 50 μL LDH reaction mixture (Beyotime, China) according to the manufacturer’s instructions. After 30 min of dark incubation at 37°C, stop solution was added per the kit manufacturer’s instructions, and absorbance at 490 nm was measured using a microplate reader. LDH release rate was calculated as [(OD_sample - OD_blank) / (OD_max - OD_blank)] × 100% (OD_max: maximum LDH release from completely lysed cells).

*Statistical Analysis*: Unless otherwise specified, all experiments were performed with at least three independent biological replicates. All quantitative data are presented as the mean ± standard deviation (SD). Statistical analyses were performed using GraphPad Prism software (version 10; Windows), with one-way analysis of variance (ANOVA) and unpaired two-tailed t-tests were applied as appropriate. A p-value < 0.05 was considered statistically significant.


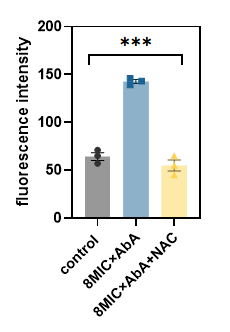
Figure S1. Rescue effect of NAC on AbA-induced intracellular ROS accumulation in *C. albicans*. The DCFH-DA fluorescence intensity in *C. albicans* under different treatment conditions.


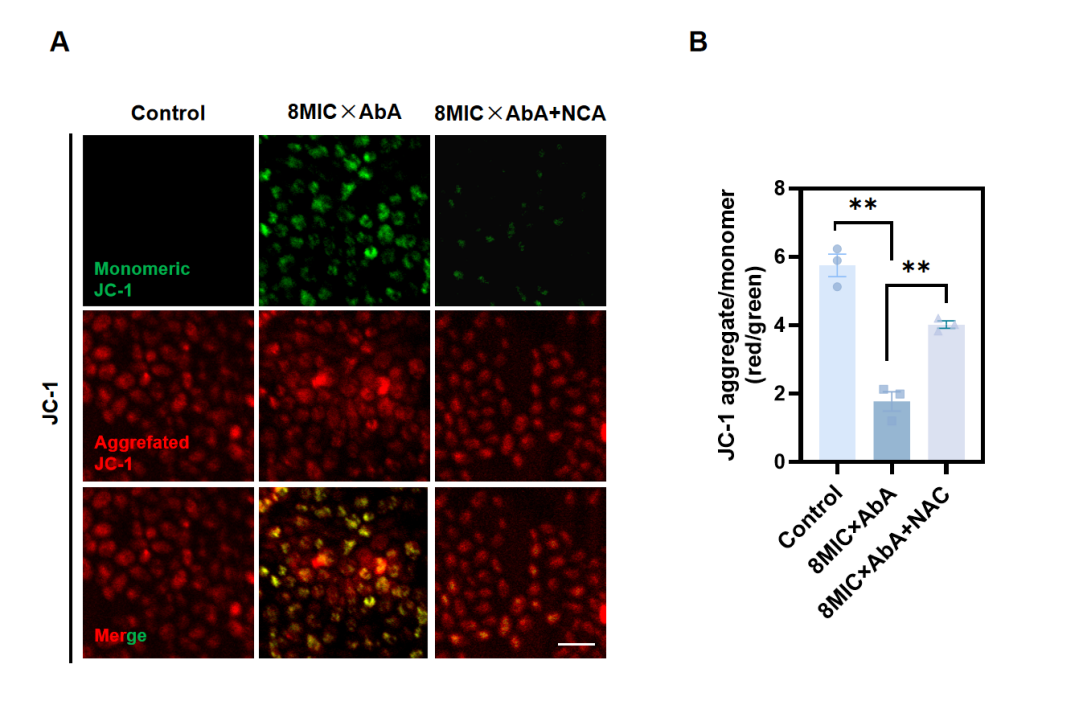
 Figure S2. NAC rescues AbA-induced mitochondrial membrane potential (MMP) reduction in C. albicans. **(A)** JC-1 fluorescence images of *C. albicans* under Control, 8×MIC AbA, 8×MIC AbA+NAC treatments. Scale bar = 5 μm; **(B)** Quantification of JC-1 aggregate/monomer ratio. Data: mean ± SD (n=3). **: p<0.01 (one-way ANOVA).


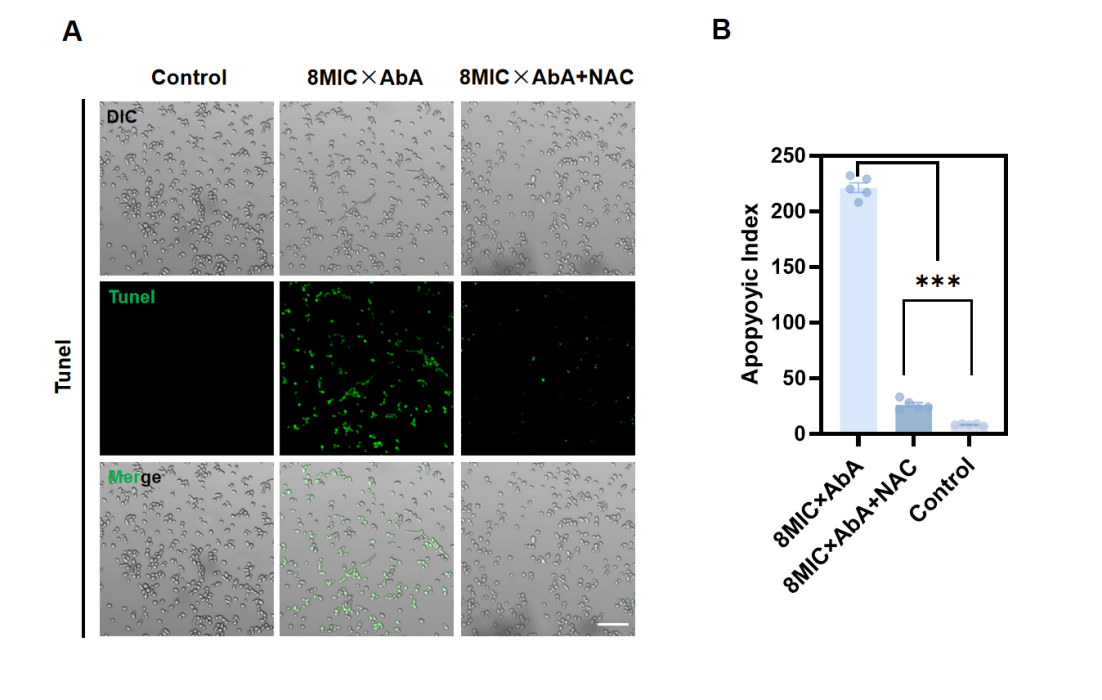


Figure S3.NAC rescues AbA-induced apoptosis in *C. albicans.* **(A)** Representative TUNEL staining fluorescent images of *C. albicans* after different treatments; scale bar=50 μm. **(B)** Quantitative analysis of TUNEL-positive cells. Data are presented as mean ± SD (n=5). ***: p<0.001 (one-way ANOVA).


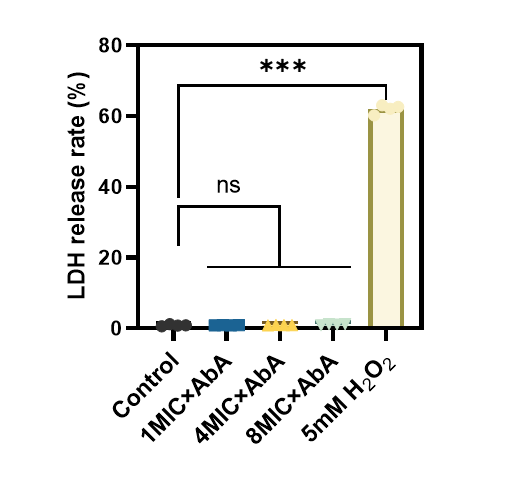


Figure S4. LDH release rate of *C. albicans* treated with different concentrations of AbA and 5 mM hydrogen peroxide. Data are presented as mean ± SD (n=4). ns:P＞0.05, ***: p<0.001 (one-way ANOVA).

**Table 1: List of primers used for RT-qPCR in this study.**

| **Primers** | **Primer sequence** |
| --- | --- |
| Tsa1-RF | F-CGGTCAAGTTCAAGGTGCTG |
| Tsa1-RR | R-CAGCAACACCACCTTGTTCC |
| Cat1-RF | F-GCTGGTGGTGTTCAAGGACT |
| Cat1-RR | R-TCCACCACCTTGTTCAGCAC |
| NADPH-RF | F-ACCAACGCCAACATCGTCTA |
| NADPH-RR | R-TGGTGGTGGTGGTGTAGTAG |
| MCA1-RF | R-F-GCTGCTGGTGCTACTGTTCT |
| MCA1-RR | R-CAGCACCAACAGCTTGTTGA |
| GADPH-RF | F-ACCACCAACTGCTTAGCACC |
| GADPH-RR | R-TCCACCACCCAGTTGTTGTT |
