# Supplementary figures and images for: ROS-implicated apoptosis in Candida albicans: mechanistic insights into Aureobasidin A's antifungal activity
Source: Front Microbiol. 2026 Feb 6;17:1725921. doi: 10.3389/fmicb.2026.1725921 (PMC12920453; doi:10.3389/fmicb.2026.1725921)

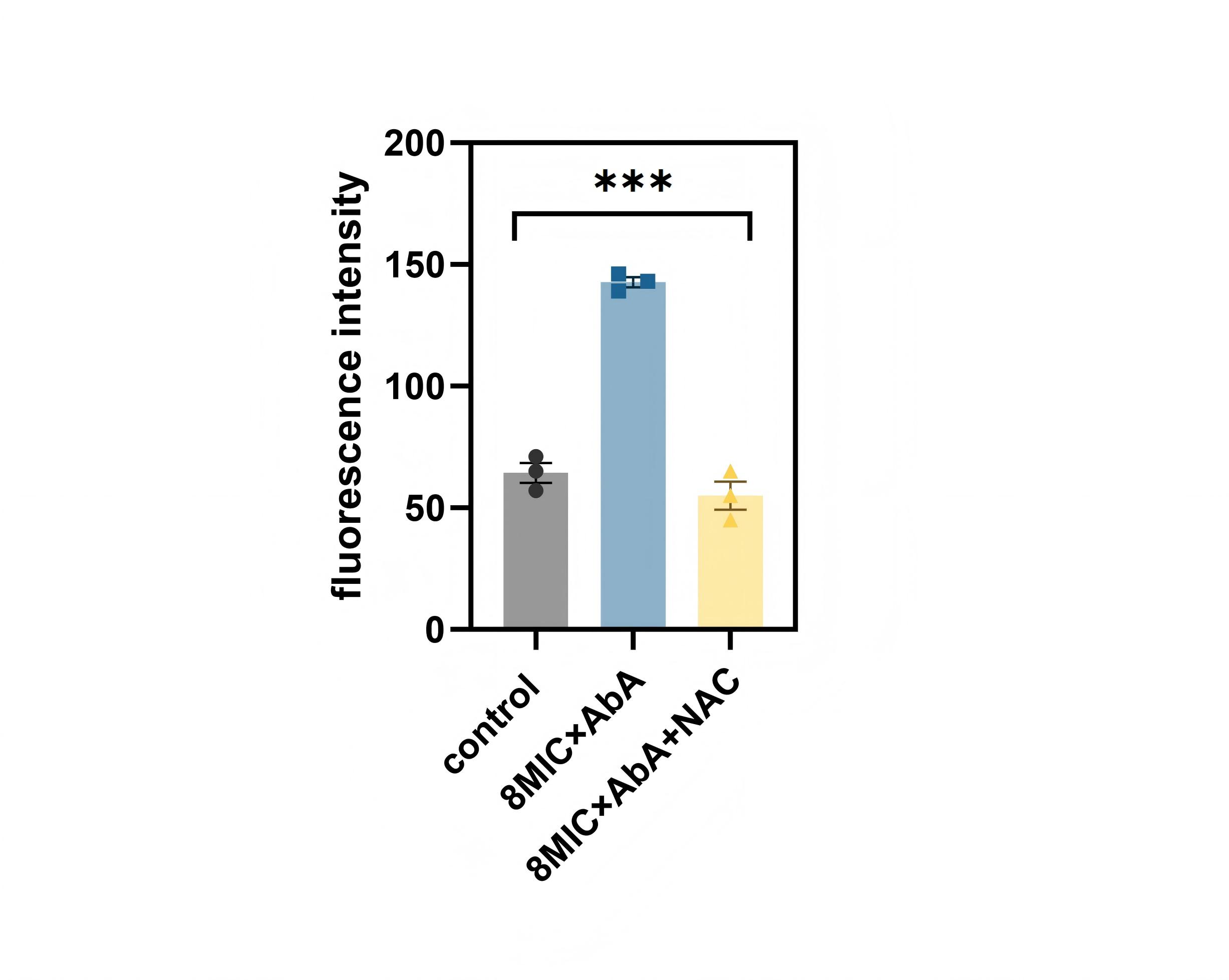

Supplement: Supplementary file 2 [file Image_1.jpeg]

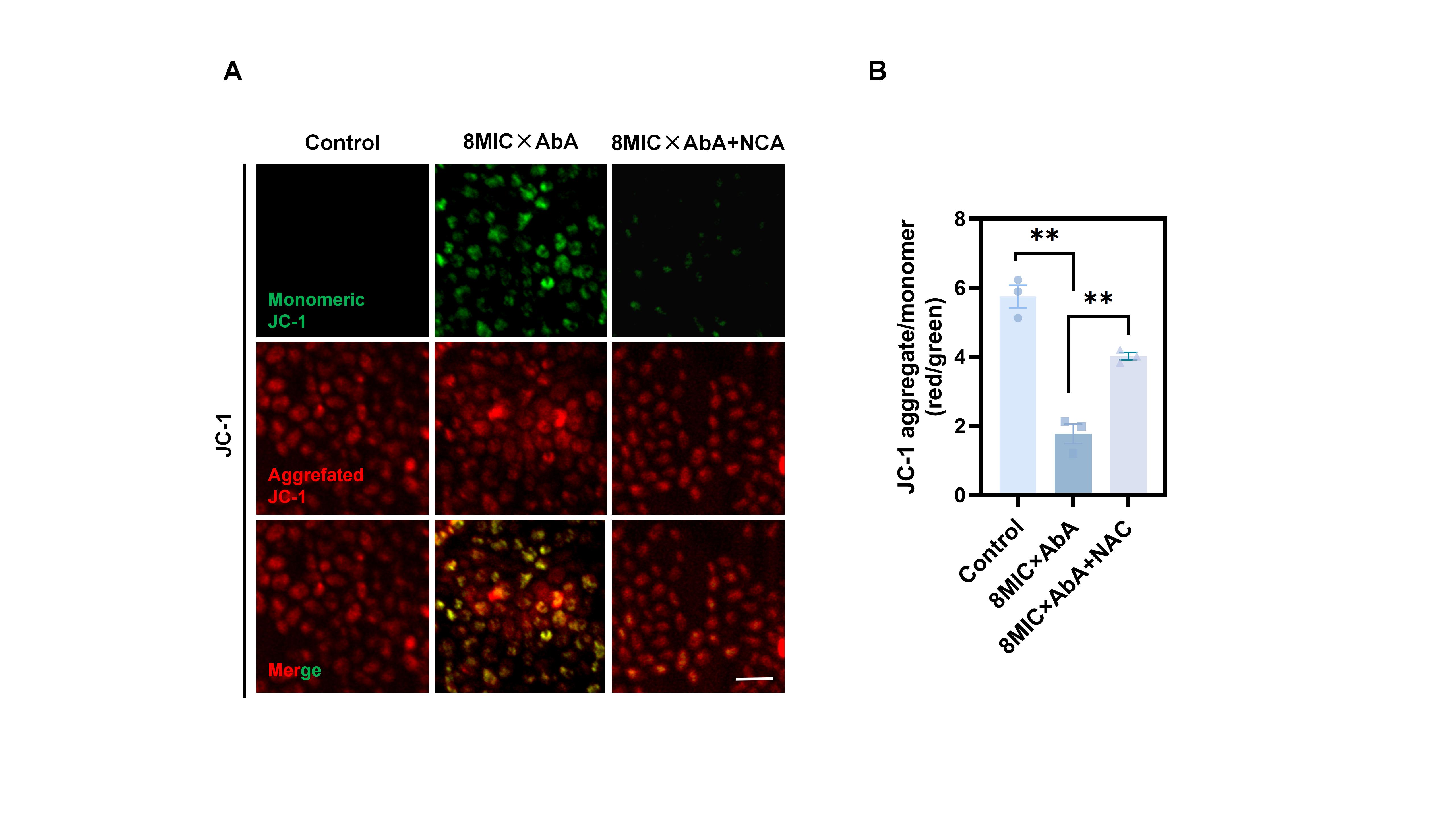

Supplement: Supplementary file 3 [file Image_2.jpeg]

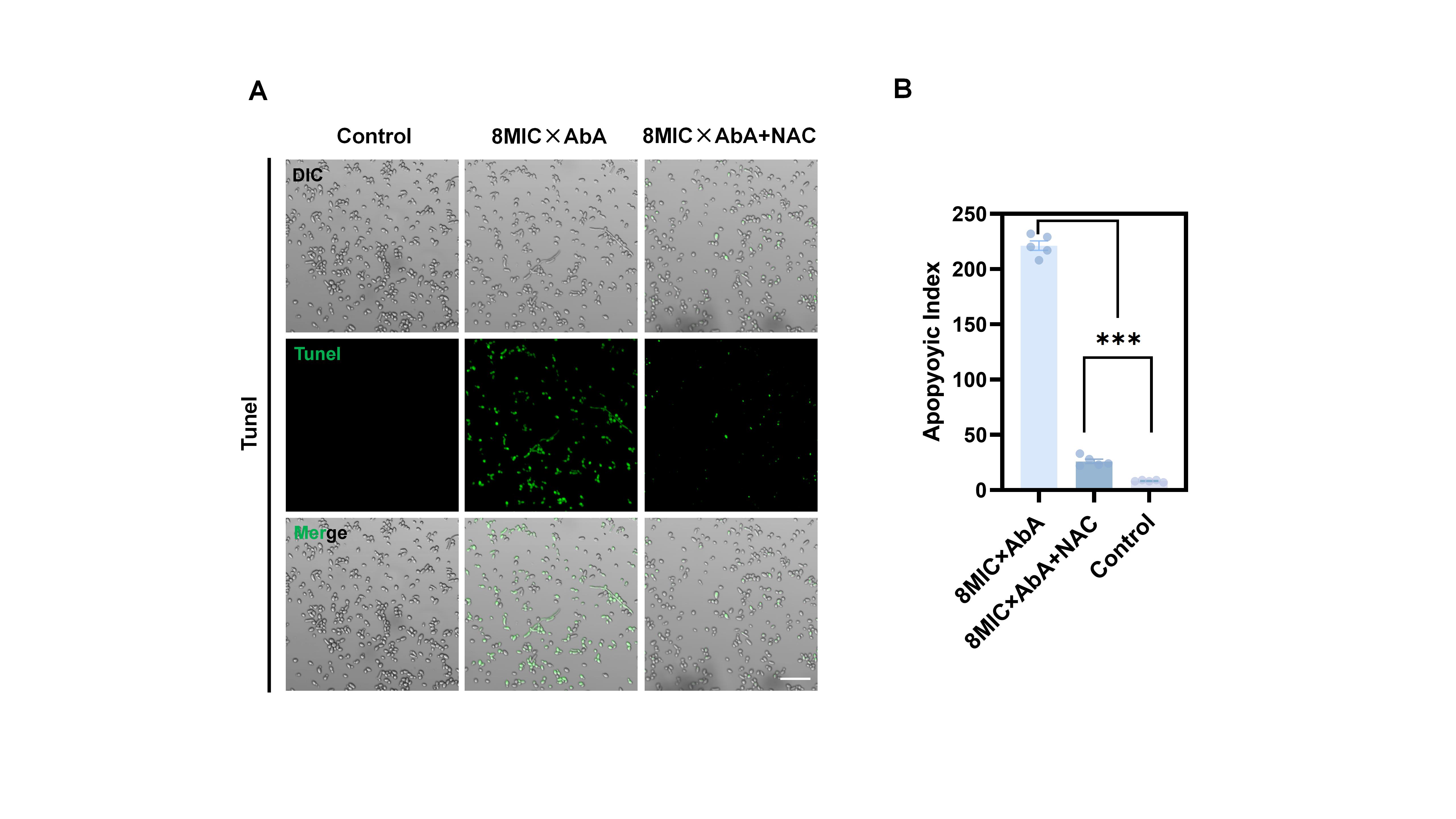

Supplement: Supplementary file 4 [file Image_3.jpeg]

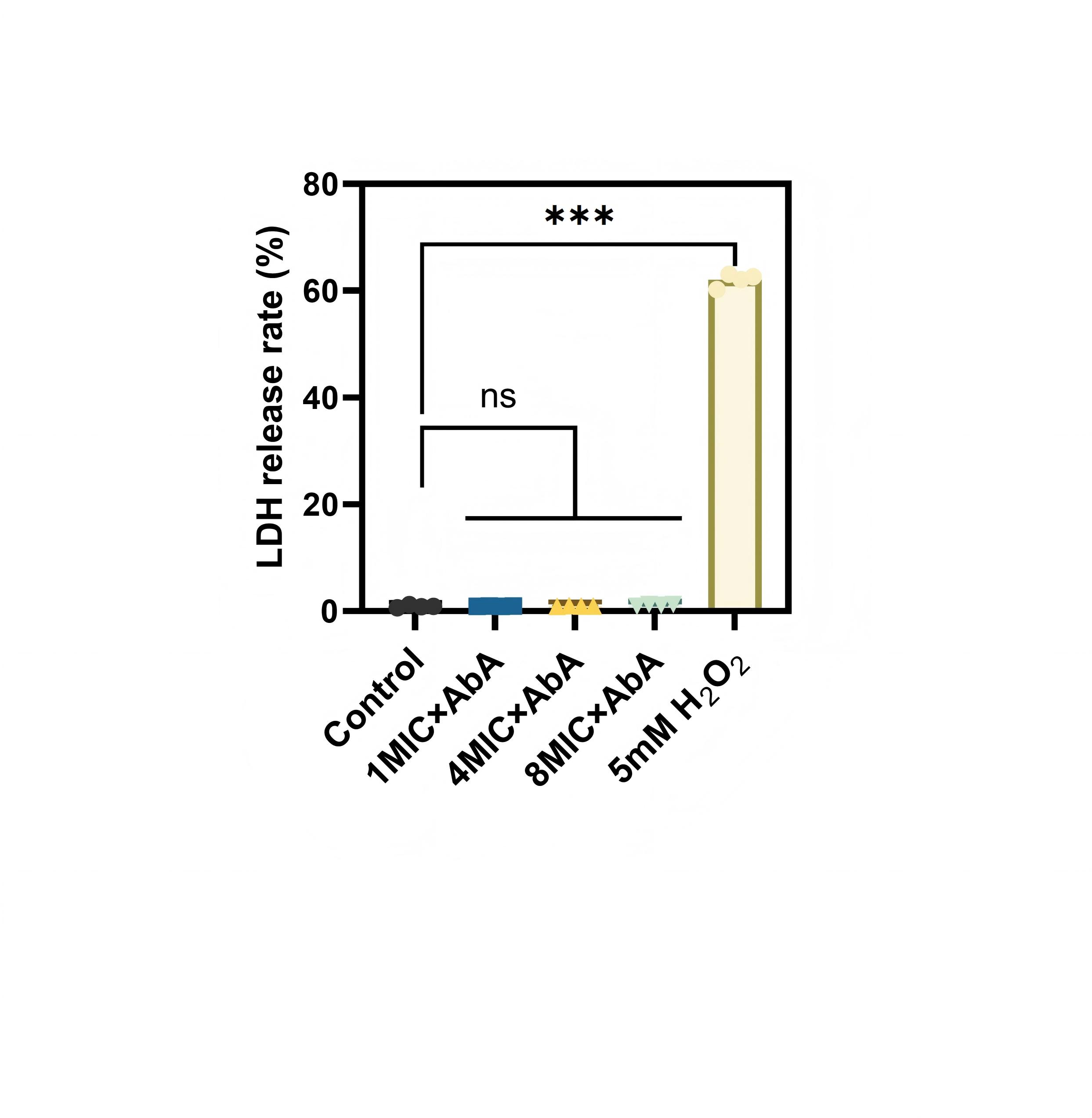

Supplement: Supplementary file 5 [file Image_4.jpeg]
